# Increased Expression of SVCT2 in a New Mouse Model Raises Ascorbic Acid in Tissues and Protects against Paraquat-Induced Oxidative Damage in Lung

**DOI:** 10.1371/journal.pone.0035623

**Published:** 2012-04-30

**Authors:** Fiona Edith Harrison, Jennifer Lee Best, Martha Elizabeth Meredith, Clare Ruth Gamlin, Dorin-Bogdan Borza, James Michael May

**Affiliations:** 1 Division of Diabetes, Endocrinology, and Metabolism, Vanderbilt University Medical Center, Nashville, Tennessee, United States of America; 2 Department of Pathology, Microbiology, and Immunology, Vanderbilt University Medical Center, Nashville, Tennessee, United States of America; National Institutes of Health, United States of America

## Abstract

A new transgenic mouse model for global increases in the Sodium Dependent Vitamin C transporter 2 (SVCT2) has been generated. The SVCT2-Tg mouse shows increased SVCT2 mRNA levels in all organs tested and correspondingly increased ascorbic acid (ASC) levels in all organs except liver. The extent of the increase in transporter mRNA expression differed among mice and among organs. The increased ASC levels did not have any adverse effects on behavior in the SVCT2-Tg mice, which did not differ from wild-type mice on tests of locomotor activity, anxiety, sensorimotor or cognitive ability. High levels of SVCT2 and ASC were found in the kidneys of SVCT2-Tg mice and urinary albumin excretion was lower in these mice than in wild-types. No gross pathological changes were noted in kidneys from SVCT2-Tg mice. SVCT2 immunoreactivity was detected in both SVCT2 and wild-type mice, and a stronger signal was seen in tubules than in glomeruli. Six treatments with Paraquat (3x10 and 3x15 mg/kg i.p.) were used to induce oxidative stress in mice. SVCT2-Tg mice showed a clear attenuation of Paraquat-induced oxidative stress in lung, as measured by F_2_-isoprostanes. Paraquat also decreased SVCT2 mRNA signal in liver, lung and kidney in SVCT2-Tg mice.

## Introduction

In most mammals vitamin C (ascorbic acid; ASC) is synthesized in the liver. In humans, non-human primates, and guinea pigs the loss of action of the *gulonolactone oxidase* gene that is responsible for the last step in ASC synthesis from glucose leads to dependence on dietary intake of ASC to avoid scurvy. ASC is transported into the blood stream, cerebrospinal fluid and cells via the action of two specific vitamin C transporters – Sodium Dependent Vitamin C Transporters 1 and 2 (SVCT1 and SVCT2) [1], [2], [3]. SVCT1 and SVCT2 are differentially distributed among organs [3]. SVCT1 is mainly localized to epithelial cells in kidney, intestines and liver. In contrast, SVCT2 is highly expressed in most other tissues including brain, lung, spleen and liver. The complete function, location and orientation of these transporters is still under investigation.

Several mouse models exist for the study of decreased levels of ASC *in vivo*. Knockout mice have been generated to study global ASC deficits due to lack of ASC synthesis (gulo(−/−) mice [4]), and for tissue specific decreases due to knockout of the two ASC transporters SVCT2(−/−) [5], and SVCT1(−/−) [6]. SVCT2(−/−) do not survive past birth and show severe hemorrhage in brain accompanying almost undetectable ASC levels in SVCT2-dependent organs ([5], [7]. SVCT1(−/−) mice have greatly increased ASC excretion and also exhibit increased perinatal mortality. In contrast to these studies on decreased ASC, and owing to the strict control exerted on ASC levels in tissues by these transporters, it is not possible to reliably increase ASC levels in tissues by oral supplementation alone [8], nor even by parenteral methods [9], although short-term increases can be seen in blood and liver following these methods. Thus, in order to study the effects of increased ASC in tissues, and to further delineate the role of ASC in response to endogenous and exogenous oxidative stress, we sought to generate a mouse model that expressed additional copies of the SVCT2. Regulation of SVCT2 and ASC levels differ among organs [10], [11] and thus an important part of the study was to assess changes in expression of the transporter and of ASC level in multiple organs. ASC is generally considered for its antioxidant benefits and so the effectiveness of increased ASC was assessed against a major oxidative stress, paraquat. However, in high concentrations ASC may also have a pro-oxidant effect [12] and so oxidative stress in the mice was also measured under baseline conditions.

To our knowledge, the only similar model currently in existence expresses additional copies of the human SVCT2 only in the eye, owing to an eye specific promoter [12]. In these hSVCT2 mice the higher quantities (up to approximately 10-fold) of both ASC and its more reactive two-electron oxidized form dehydroascorbic acid (DHA) in the lens contributed to protein damage via the Maillard reaction. A number of advanced ascorbylation productions were increased in the lenses of transgenic animals. Lenses were also noted to have taken on yellow brown coloring similar to that typically observed in the aging human lens. In the present study we sought to test the effect of systemic increases in SVCT2 and whether any resultant increase in ASC levels would be protective against oxidative insult or would contribute to oxidative damage as in the Fan et al. studies. Thus, the overall aim of these experiments was to generate a mouse that had global increases in SVCT2 expression and document the concomitant changes in ASC. We further sought to whether increased tissue ASC would protect against paraquat-induced oxidative stress. Data supportive of both of these aims suggest that the SVCT2-Tg mouse will be a useful model for the study of ASC transport and oxidative stress processes.

## Materials and Methods

### Ethics Statement

All procedures were approved by the Vanderbilt University committee for Institutional Animal Use and Care and were conducted in accordance with NIH guidelines.

### Generation of Slc23a2 Transgenic Mouse

The SVCT2 BAC clone, RP24-222C8, containing the 60kb gene for *slc23a2* and 90kb surrounding DNA was selected from Clone Finder and purchased from Invitrogen. It was selected because the sequences at its ends placed it in a region of the mouse genome free of other genes. It contained ∼50,000 bases preceding the gene and so it was likely to have most or all of the promoter sequences. The purified DNA (1 ng/µl) was microinjected into the pronuclei of one-cell 12 h old B6D2F1 mouse embryos in the Transgenic Mouse/Embryonic Stem cell shared resource core of the Vanderbilt Ingram Cancer Center. Two chimeric founder mice were obtained as determined by genotyping the progeny for BAC vector sequence (Table 1). Two of these lines showed increased copy number of *slc23a2* as determined by qrtPCR with actin as a reference gene. The line expressing higher levels of SVCT2 was chosen and maintained as a colony. All mice used in this study were back-crossed at least 9 times onto the C57Bl6 background (Jackson labs).

**Table 1:** Primer sequences used for PCR.

Target	Sequence
	**Primers used for genotyping PCR**
SVCT2	CAT CTG TGC GTG CAT AGT AGC
	CAC CGT GGC CCT CAT TG
	TCT GAG CCC AGA AAG CGA AG
	GAT GGACGG CAT ACA AGT TC
SVCT2-Tg (BAC)	Forward: GGAAATCGTCGTGGTATTCACTC
	Reverse: TCCCAATGGCATCGTAAAGAAC
	**Primers used for RT-PCR**
Slc23a2 for SVCT2-Tg	Forward: AAGGATGGACGGCATACAAG
	Reverse: TCTGTGCGTGCATAGTAGCC
β-actin	Forward: GTTTGAGACCTTCAACACCCC
	Reverse: GTGGCCATCTCCTGCTCGAAGTC
SVCT1	Forward: CAATATAGAAACTGGGTCTGTGTG
	Reverse: CCAACTCAGGTCTTCTGTCTC

### Slc23a2 Knock Out Mice

Mice heterozygous for a targeted (lethal) knockout of *slc23a2* (SVCT2-KO(−/−)) were originally obtained from Dr. Robert Nussbaum and are maintained as a colony in house. Homozygous SVCT2-KO(−/−) mice survive the gestation period but do not survive past birth and die without breathing and showing extensive hemorrhage in the brain [5], [7]. The presence of the knockout gene was detected using a primer set which amplifies DNA spanning the native DNA and the neomycin resistance gene belonging to the substituted DNA (Table 1). Heterozygous mice (SVCT2-KO(+/−)) were crossed with SVCT2-Tg mice to determine functionality of the transgene by ability to rescue ASC levels and promote survival in SVCT2-KO(−/−) mice.

### SVCT2-Tg Mice used in Studies

Initial studies of SVCT2 expression and rescue of the SVCT2-KO(−/−) genotype were performed with hemizygous mice. Later lines were maintained as homozygous transgenic mice. For all other studies reported below, adult SVCT2-Tg mice (3–6 months) were used. Mice were bred from homozygous SVCT2-Tg males and females and offspring were checked for presence of the BAC gene, indicating additional copies of SVCT2. Expression amounts varied between mice, and even among tissues in the same mouse, so expression was determined post-mortem by RT-PCR as described below. Group numbers are reported for each experiment. The breeding schedule did not permit use of littermates as controls, instead we used age-matched wild-type mice from the background strain C57Bl6 that were maintained as a colony in-house.

### mRNA Extraction and Real Time PCR

Total RNA was extracted using GenEluteTM Mammalian Total RNA Miniprep Kit (Sigma–Aldrich). Tissue samples were homogenized with an electric homogenizer and total RNA was extracted according to the manufacturer’s protocol. RNA concentrations were determined relative to protein by measuring absorbance at 260 and 280 nm, respectively. cDNA was synthesized from RNA samples using TaqMan Reverse Transcriptase (Sigma–Aldrich) following the manufacturer’s protocol. Real time PCR (qrtPCR) was then performed to establish transporter copy number for Slc23a2 and β-actin. Primers used are shown in Table 1. SybrGreen (BioRad) reagent was used with the MyIQ apparatus (BioRad).

### Fluorescence in Situ Hybridization

Fluorescence in Situ Hybridization (FISH) was performed as a service by the Van Andel institute Laboratory of Germ line Modification and Cytogenetics in Grand Rapids, Michigan. FISH was performed on a descendent of the transgenic line used in this study. Chromosome pairs were identified using spectral karyotyping (SKY).

### Measurement of Ascorbic Acid

ASC content of tissues, serum, and urine was determined by HPLC according to previously published methods [9], [13], [14]. Briefly, weighed tissue samples were homogenized sequentially in a combination of two solutions, 25% (w/v) aqueous metaphosphoric acid and 100 mM sodium phosphate buffer containing 5 mM EDTA, with a final ratio of 2∶7. Serum was diluted 1∶9 with 200 mM sodium perchlorate, and urine was diluted 1∶20 with 90% methanol with EDTA. Samples were centrifuged and the supernatant was taken for assay of ASC as described above following appropriate dilution with HPLC mobile phase.

### Measurement of Malondialdehyde (MDA)

MDA was quantified as thiobarbituric acid reactive substances (TBARS) according to previously published methods [15] as a measure of lipid peroxidation. Briefly, weighed samples were homogenized in 1 ml 5% trichloroacetic acid. Samples were centrifuged and 250 µl of the supernatant was reacted with the same volume of 20 mM thiobarbituric acid for 35 min at 95°C, followed by 10 min at 4°C. Sample fluorescence was read using a spectrophotometric plate reader with an excitation wavelength of 515 nm and emission wavelength of 553 nm.

### Measurement of F_2_-isoprostanes

F_2_-isoprostanes were measured in the Vanderbilt Eicosanoid core by gas chromatography/negative ion chemical ionization mass spectrometry (GC/NICI-MS) employing stable isotope dilution with [^2^H_4_]-15-F_2t_-IsoP as the internal standard [16].

### Kidney Function

Urinary albumin excretion was measured in spot urine samples by capture ELISA using a mouse albumin quantitation kit (Bethyl, Montgomery, TX). Urine creatinine was measured using Infinity creatinine stable reagent (Thermo Fisher Scientific, Middletown, VA), according to manufacturer’s protocols. Albuminuria was expressed as urinary albumin to creatinine ratio (ACR). Portions of mouse kidneys were fixed in buffered formalin, dehydrated through graded ethanols, embedded in paraffin, and sections (2 µm thick) were stained with hematoxylin and eosin (H&E). Additional kidneys were taken from different SVCT2-Tg and wild-type control mice and frozen in OCT medium prior to preparation of 5 µm sections using a cryostat. Each section was stained for SVCT2 (primary: goat Abs to SVCT2, S-19, Santa Cruz cat. #sc-9926, diluted 1∶150; secondary: donkey anti-goat Abs Alexa Flour 488 (cat. #A11055, diluted 1∶1000) and von Willebrand factor 8 (primary: rabbit Abs to vWF, diluted 1∶500; secondary: donkey anti-rabbit Abs Alexa Fluor 594 (cat. #A21207, diluted 1∶1000)). Von Willebrand factor 8 was used to stain for endothelial cells that lines capillaries in the glomerulus. Staining for SVCT2 was also performed without incubation with primary antibody to confirm specificity of staining.

### Behavioral Analysis

A series of behavioral tests were conducted with 3-month-old SVCT2-Tg (5 male and 5 female) and wild-type (5 male and 5 female) mice in order to establish whether there were any behavioral consequences of increased SVCT2 expression.

Mice were first tested on the *Elevated Zero Maze (EZM)* for anxiety as indexed by exploration of open and enclosed areas (5 min. trial, data analyzed using AnyMaze (Noldus)). *Locomotor activity* was measured by infra-red beam breaks in standard activity chambers (32 cm^2^, Med PC Associates) during two 15 min. trials on days 1 and 3. Repeated measurements permitted measurement of anxiety (time spent in center versus edge of chamber on initial trial) and also of habituation to the chambers (memory for testing environment) indexed through decreasing activity across time and between the sessions. Alternation behavior in the *Y-maze* was used as a measure of spatial working memory. Mice were permitted to explore the Y-shaped maze for 5 min. and the number and pattern of arm entries were noted by the experimenter. An arm entry was counted when the whole mouse entered the arm, and an alternation was designated as entry into each of the three arms within three consecutive arm entries (i.e. ABC, but not BAB). Percent alternation was calculated as alternations/(total entries –2). Neuromuscular ability was tested on the *rotorod* in repeated training sessions on three consecutive days. Three trials, up to a maximum of 300 s per trial were administered during each training session with an inter-trial interval of between 5 to 15 mins. During the trial, the mice attempted to remain balanced on an accelerating rotorod (4 rpm up to a maximum of 40 rpm). Time to the first rotation (grasping the rod and rotating along with it instead of walking on top of it) and time to fall were noted by the experimenter.

### Treatment with Paraquat

Mice were treated with paraquat (PQ, paraquat dichloride, Sigma, USA) in order to induce oxidative damage by elevated levels of superoxide [17]. Mice were injected with three treatments of 10 mg/kg followed by three further treatments of 15 mg/kg (intraperitoneal, administration volume 10 ml/kg). This treatment was designed to induce a chronic low level of damage. Treatments took place on days 1, 4, 9, 12, 15, & 18 and mice were sacrificed and tissues removed for analysis on day 19 of the study. Mouse health was monitored daily and mice were weighed frequently during the treatment period. Control mice were treated with the vehicle alone, which was 0.9% saline. Following previous reports that PQ treatment can affect activity levels and sensorimotor abilities [18], mice were tested in the locomotor activity chambers and on the rotorod according to methods described above. Locomotor activity testing was conducted across a single 30-min. session, 20-min. after PQ or saline treatment on day 15. Rotorod testing took place 3 hours following treatments on day 15, and on day 16 (non-treatment day). Mice included in this study were (PQ N = 5 wild-type, N = 5 SVCT2-Tg; Saline N = 5 wild-type, N = 5 SVCT2-Tg).

### Statistical Analyses

#### Biochemical data

Biochemical data were analyzed in Prism 5 for Mac by unpaired t-test (2-tailed) with separate analyses conducted for each tissue type in each assay. In cases where equal variance in data could not be assumed, Welch’s correction for unequal variances was applied to analyses. ASC and MDA determination were made with two separate cohorts of mice, therefore, to ensure consistency within the results, data from the second cohort were transformed using z-scores to normalize them with reference to the original cohort. The same transformation was not required for mRNA data for which each data from each cohort were already expressed relative to wild-type. Transgene copy number varied among mice and among tissues within each mice, therefore, we have provided scatterplots showing data points from each individual mouse for each measure rather than group means.

#### Behavioral data

Behavioral data were analyzed with SPSS Version 19 for Mac. For single measure tests (Y-maze, EZM, open field), univariate ANOVA was used. In cases of repeated testing across trials (locomotor activity, rotorod) repeated measures ANOVA was used. Data were first analyzed with both genotype (SVCT2-Tg; wild-type) and gender (male; female) as between-groups factors. If there was no main effect of gender, then data were collapsed across gender and analyses were repeated with genotype as the only between groups factor. Where there was a main effect of gender data are reported for male and female mice separately.

## Results

### Generation of Founder Mice

After gestation in pseudopregnant females one founder mouse was obtained. This male (Bac2) expressed a 2.2±0.2-fold increase in SVCT2 genomic DNA by real-time PCR relative to wild-type littermates and was used to generate the SVCT2-Tg colony used in this experiment. The founder was bred to C57Bl/6 mice for at least 10 generations to place the transgene on this background before experimentation began.

### Location of Transgene

**Figure 1:**
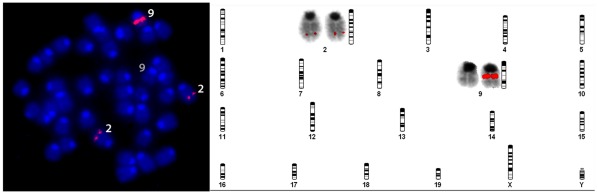
Fluorescent In Situ Hybridization for SVCT2. FISH analysis localized the SVCT2 transgene to one copy of chromosome 9 (#9), whereas the native version was found on both copies of chromosome 2 (#2). Brighter signal indicates multiple copies of the transporter at the insertion site.

FISH was used to determine the location of the transgene in one of the lines. The BAC transgene that was passed in the germ line was found on chromosome 9, while the native gene is on chromosome 2 (Fig. 1). The BAC transgene was also observed to be much brighter than the native copies, indicating higher expression of the transgene than native copies.

### Rescue of the Lethal Phenotype by the Transgene

In order to determine if the SVCT2 transgene could substitute for the native gene, it was necessary to show two copies of the ablated native gene in a surviving transgenic mouse. Matings between SVCT2-Tg and SVCT2-KO(+/−) heterozygous animals were carried out. From the progeny SVCT2-Tg.SVCT2-KO(+/−) were selected and bred together. When PCR was performed on genomic DNA of the pups born to these parents, four out of 18 surviving pups were confirmed as homozygous knockouts (SVCT2-KO(−/−)) that also carried the BAC transgene. SVCT2(−/−) do not normally survive past birth indicating that pup lethality was rescued by the transgene. In a final crossing, an SVCT2-Tg.SVCT2-KO(−/−) mouse was bred to an SVCT2(+/+) wild-type mouse, 23 pups sired by this male were all heterozygous SVCT2-KO(−/+) confirming that the genotype of the sire was SVCT2-KO(−/−), which is the normally lethal genotype.

### Normal Post-natal Development

Pups born to two SVCT2-Tg parents were normal in appearance. Mice from ten litters of SVCT2-Tg pups (average litter size 5.5) and seven wild-type pups (average litter size 5.29) were weighed every 3 days from birth to 21 days. All mice increased in mass with age (p<0.001) but there was no main effect of genotype (p = 0.09; data not shown). Litter size was not different from wild-type mice (p = 0.86).

### ASC and mRNA

**Figure 2:**
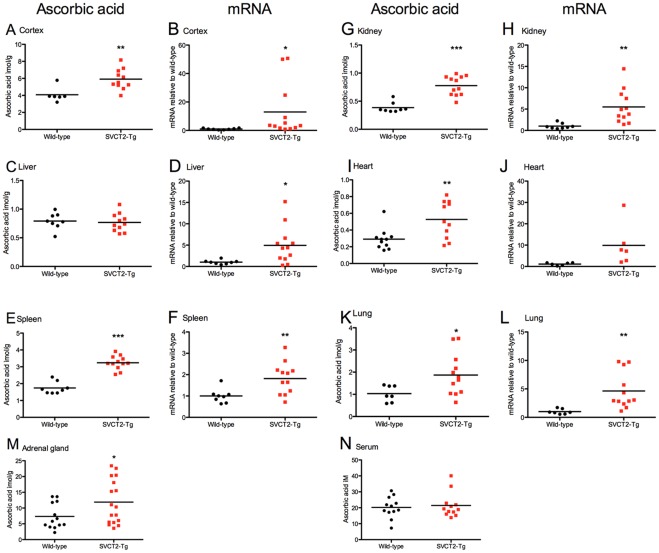
Ascorbic acid and mRNA level in SVCT2-Tg mice. SVCT2-Tg had higher ASC levels in all organs measured except liver, nor did serum ASC vary between groups. (A) cortex, (C) liver, (E) spleen, (G) kidney, (I) heart, (K) lung, (M) adrenal gland, and (N) serum. mRNA signal was increased in all organs measured, although this difference was not significant in heart. (B) cortex, (D) liver, (F) spleen, (H) kidney, (J) heart, (L) lung, (M) adrenal gland, and (N) serum. *, **, *** p<0.05, 0.01, 0.001 compared to wild-type controls.

Intracellular vitamin C levels are tightly controlled, so that it is difficult to increase them by dietary intervention in mice that synthesize their own ASC. Expression of additional SVCT2 in the transgenic animals led to increases in ASC in organs of these mice. These increases were significant in brain cortex, spleen, kidney, heart and lung (p<0.05, Fig. 2A, E, G, I & K). In adrenal glands increased ASC was seen in some of the SVCT2-Tg mice from the first cohort in a similar pattern to other tissues described above. However, these increases were not significant, possibly due to lower group size or small size of tissue sample for adrenal glands and possible contamination of samples with adipose tissue. When additional samples were added to the sample from a separate cohort of mice, significant differences were found between the two genotypes (p<0.05, Fig. 2M). No differences were seen between the groups in serum or liver ASC levels (Fig. 2C & N). Both wild-type and transgenic mice are capable of synthesis of ASC in liver and some regulation of synthesis may have occurred to maintain these levels at normal.

The same tissues were assayed for mRNA expression to determine the effect of the transgene insertion on expression of SVCT2 mRNA. Increased SVCT2 mRNA compared to wild-type levels was found in each of the tissues studied in some or all of the transgenic mice leading to overall group increases in cortex, liver, spleen, kidney, and lung (Fig. 1B, D, F, H & L). Fewer heart samples were available for mRNA expression and the increased copy number seen in this tissue was not significantly greater than that seen in wild-types (p = 0.089). The extent of the increase in mRNA expression varied between mice and between organs. Adrenal gland was not studied owing to small sample size.

Of particular interest is that SVCT2 mRNA expression level and tissue ASC level were related in some but not all tissues. Significant correlations between mRNA level and ASC concentration in the same mice were found in spleen (r = 0.585, p<0.01), lung (r = 0.496, p<0.05), and kidney (r = 0.687, p<0.001). The relationships between mRNA level and ASC were also positive, but not significant in cortex, liver, and heart (p>0.11).

### Oxidative Damage in SVCT2-Tg Mice

**Figure 3:**
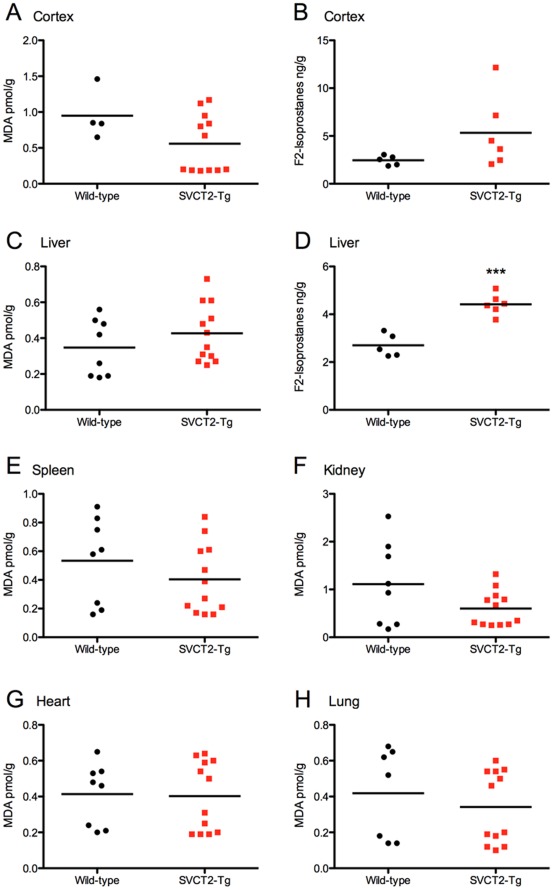
Oxidative stress in SVCT2-Tg mice. Oxidative stress was quantified as malondialdehyde (TBARS) in (A) cortex, (C) liver, (E) spleen, (F) kidney, (G) heart, and (H) lung, and as F_2_-isoprostanes in (B) cortex, and (D) liver). *** p<0.001 compared to wild-type controls.

We first quantified MDA as TBARS in tissues from SVCT2-Tg and wild-type mice to establish whether additional ASC in tissues could act as a pro-oxidant. We found no differences in MDA level in the majority of the tissues tested, and in fact there was the suggestion of a protective effect of ASC in the kidney (Fig. 3 A, C, E-H). However, MDA levels were slightly, but not significantly, increased in the liver of SVCT2-Tg mice. In order to further quantify oxidative damage in these mice using a more specific measure of lipid peroxidation of arachidonic acid, we quantified F_2_-isoprostanes in brain and liver of six SVCT2-Tg and five wild-type mice. An apparent increase in cortex F_2_-isoprostanes in SVCT2-Tg mice was mostly driven by two of the six mice and the difference was not significant (p = 0.13, Fig. 3B). We verified the ASC and SVCT2 mRNA levels of these mice to establish whether there was a direct relationship between SVCT2 expression and F_2_-isoprostanes level. However, in the cortex the ASC and mRNA levels were within the middle range group and were actually within normal wild-type levels. A more modest but less variable increase in F_2_-isoprostanes in the liver was significant (p<0.001, Fig. 3D), supporting the result found with MDA.

### SVCT1

To assess whether compensatory mechanisms may be in place to explain the lack of change of ASC in the liver under baseline and PQ-treatment conditions, we assessed SVCT1 mRNA levels. SVCT1 expression was also studied in the kidney, which also expresses both SVCT1 and 2, but which showed increased ASC in SVCT2-Tg mice. Neither liver nor kidney differed in SVCT1 mRNA expression according to genotype (p>0.17, data not shown).

### Kidney Function

**Figure 4:**
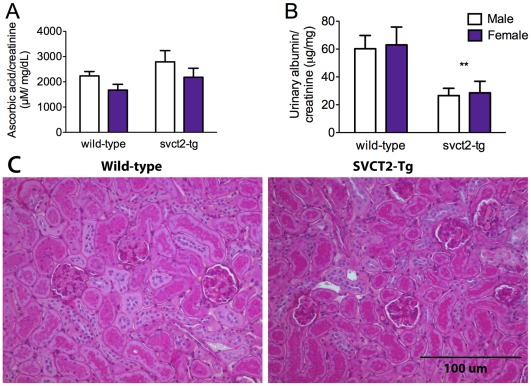
Kidney Function in SVCT2-Tg mice. Excreted (A) ASC and (B) albumin were measured in urine samples with the results normalized to creatinine. Data shown are mean+S.E.M for wild-type and SVCT2-Tg mice separately for male (white bars) and female (purple bars) mice. ** p<0.01 compared to wild-type control. N = 5−6 mice per group. (C) 2 µm sections stained with hemotoxylin and eosin (H&E) for comparison between genotypes. Glomeruli, proximal tubules and distal tubules are all clearly visible but no changes were observable in SVCT2-Tg mice (right panel) compared to wild-types (left panel). Images taken at 20X magnification.

ASC metabolism is generally well controlled in the body with excess ASC being excreted in the urine. Reabsorption of ASC in the kidney is the responsibility of SVCT1. SVCT2-Tg mice have higher organ levels of ASC, but not higher plasma ASC. We therefore determined ASC excretion to ascertain whether additional excretion of ASC was a mechanism by which the mice were regulating circulating levels. ASC excretion was calculated relative to creatinine to account for any difference in mouse water intake prior to the sample acquisition. As has previously been reported [6] female mice excreted less ASC than male mice, although this difference was not significant in this study (Fig. 4A). There was no difference between wild-type and SVCT2-Tg mice. However, urinary albumin excretion was significantly lower in SVCT2-Tg mice of both genders (p<0.01; Fig. 4B). Although both wild-type and SVCT2-Tg mice were within the normal range for albumin in the urine, this result suggests potentially improved kidney function in the SVCT2-Tg mice.

**Figure 5:**
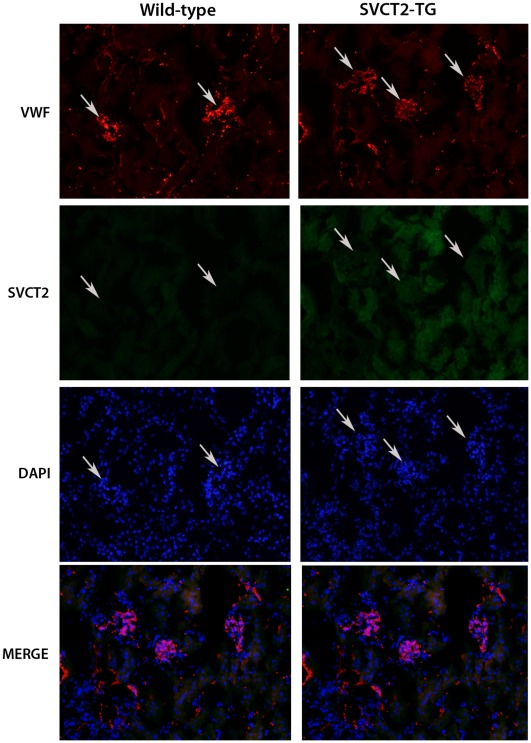
SVCT2 location in kidney. 5 µm frozen kidney sections were stained for von Willebrand factor (VWF, red) and for SVCT2 (green) with non-specific stain for nuclei (blue) in wild-type (left panels) and SVCT2-Tg (right panels) kidneys. Heavier staining for VWF is observed in glomeruli (arrows) with corresponding areas of low staining for SVCT2 and high density of nuclei. A merged image (bottom panels) confirms lack of overlap of SVCT2 and VWF in glomeruli. Images taken at 20X magnification.

Careful analysis was made of H&E stained kidney sections from male SVCT2-Tg and wild-type mice by an experienced renal pathologist. No evidence of any gross pathological differences was found in the SVCT2-Tg mice (Fig. 4C). Additional frozen sections of kidney from a different set of mice were stained with antibodies for vWF and SVCT2 for localization of the SVCT2. Glomeruli can be clearly seen as areas of greater staining for vWF (Figure 5, arrows) with dense nucleation in both wild-type and SVCT2-Tg mice. SVCT2 staining did not co-localize with vWF and was higher in non-glomerulus areas, suggesting its presence in tubules. SVCT2 mRNA and vitamin C level were confirmed to be higher than wild-type in the SVCT2-Tg mice used for fluorescent immunohistochemistry (1.94, 6.97, and 7.70 fold wild-type expression; wild-type ASC 0.61+0.17, SVCT2-Tg 1.60+0.31 µmol/g). Staining was not formally quantified, however, no differences in signal strength or distribution were noted between wild-type and SVCT2-Tg mice.

### Behavioral Phenotype

**Figure 6:**
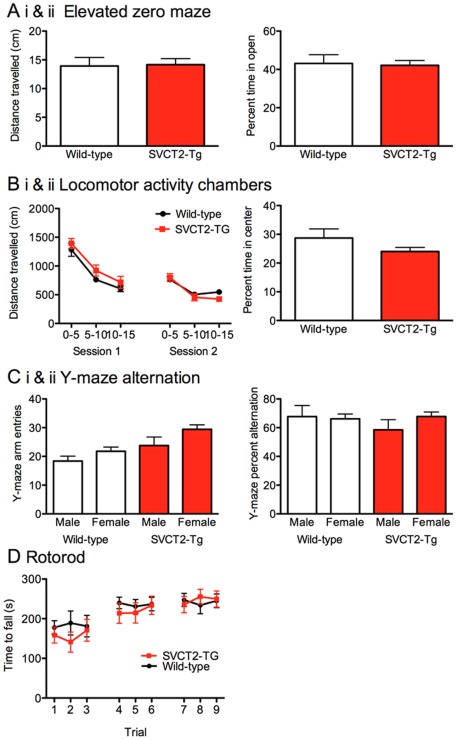
Normal behavior in SVCT2-Tg mice. Wild-type (white bars, black circles) and SVCT2-Tg mice (red bars, red squares) did not differ on any of the behavioral measures conducted. (A) Elevated zero maze for (Ai) distance travelled and (Aii) percent time in open zones, (B) locomotor activity (Bi) time traveled during two 15-min. sessions, and (Bii) time spent in the central portion of the maze during the first 5-min., (Ci) entries and (Cii) alternation in the Y-maze, and (D) time taken to fall or rotate on the rotorod. Data shown are mean+S.E.M. N = 10 mice per group.

Behavioral data are reported for 10 mice of each genotype with equal numbers of male and female mice in each group. SVCT2-Tg mice were normal compared to wild-type controls on each of the measures. Exploration of the EZM (distance travelled) and percent time spent in open areas did not differ between the genotypes or according to gender (ps>0.077; Fig. 6Ai, ii). Distance traveled in locomotor activity chambers decreased across the 5-min. time bins within each 15-min. session (p<0.001) and between the two test sessions (p<0.001), but did not vary according to genotype or gender (ps>0.56; Fig. 6Bi) indicating normal locomotor activity and habituation processes in the SVCT2-Tg mice. Locomotor activity data were also analyzed to establish time spent and distance travelled in the center portion of the chamber versus the edge and corners during the first 5 minutes in the novel environment as a further measure of anxiety (open field measurement). The center portion comprised 46% of the total area of the chamber wild-type and SVCT2-Tg mice spent only 24% and 28% of their time respectively in the center portion. Neither time spent in center nor distance travelled in center varied according to gender or genotype (ps>0.195; Fig. 6Bii). In the Y-maze, female mice made more arm entries than male mice (p<0.05) and SVCT2-Tg mice made more entries than wild-type mice (p<0.01; Fig. 6Ci) but all mice performed within the normal range for this test. There was no interaction between gender and genotype (p = 0.591). Despite varying amounts of exploration in the maze, alternation behavior did not vary according to genotype or gender (ps>0.36; Fig. 6Cii) indicating intact spatial working memory in the SVCT2-Tg mice. All mice had normal sensorimotor abilities and learned to perform well on the rotorod, as shown by increasing trial times across the three test sessions (ps<0.001; Fig. 6D). There were no effects of gender or genotype on performance in time to first rotation or to fall (ps>0.233).

### Response to Paraquat Treatment

To investigate the possibility of a protective role of elevated ASC in response to elevated superoxide levels, mice were treated with paraquat. Continuous monitoring during the treatment time revealed very little loss of weight in the treated mice (p>0.15, data not shown). Following previous reports that PQ treatment can affect activity levels and sensorimotor abilities, mice were tested for changes in locomotor activity and sensorimotor abilities on the rotorod. All mice showed expected habituation over time in the activity chambers (p<0.001), but there was no main effect of group and no group X time bin interaction (ps>0.21). In contrast to earlier reports [18], neither task revealed any effect of PQ, and neither was there any difference in response between the genotypes (data not shown).

**Figure 7:**
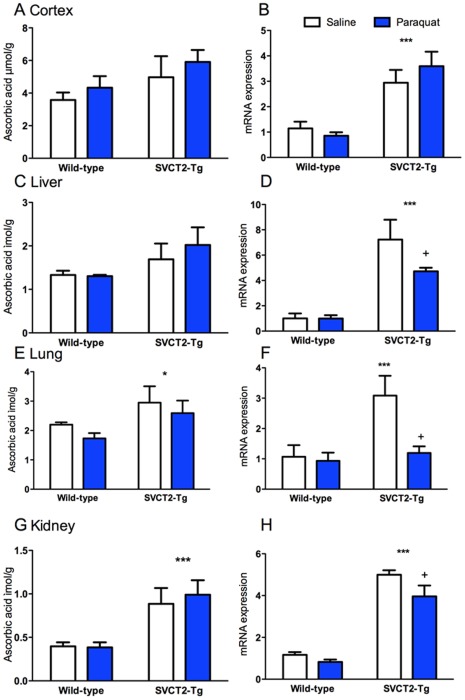
Ascorbic acid and mRNA levels following paraquat treatment. ASC was increased in (A) cortex, (C) liver, (E) lung, and (G) kidney, although this difference was only significant in lung and kidney. SVCT2 mRNA was significantly higher in SVCT2-Tg mice in (B) cortex, (D) liver, (F) lung, and (H) kidney, but significant decreases were found in PQ-treated mice compared to saline treated SVCT2-Tg mice. *, *** p<0.05, 0.001 different from wild-type control; +PQ-treated mice different from saline-treated mice of same genotype. Data shown are mean+S.E.M for wild-type and SVCT2-Tg mice treated with saline (white bars) and paraquat (blue bars). N = 5 mice per group.

ASC and mRNA were measured in the four organs thought most likely to be affected by PQ treatment. In contrast to earlier results, ASC levels were higher in SVCT2-Tg mice than wild-types in the cortex but this difference was not significant (p = 0.097; Fig. 7A). As reported above, neither were liver ASC levels significantly greater in SVCT2-Tg mice (p = 0.067; Fig. 7B). ASC levels were increased as expected in lung and kidney of SVCT2-Tg mice (ps<0.05; Fig. 7E, G). Analysis of SVCT2 mRNA revealed some unexpected differences among the tissues. In the brain there were higher levels of SVCT2 mRNA in the SVCT2-Tg mice (P<0.001), and no effect of PQ (p = 0.66; Fig. 7B). In the liver, lung and kidney, there was a significant main effect of genotype on mRNA expression in each organ (ps<0.05; Fig. 7D, F & H). There were also significant main effects of PQ treatment in kidney and lung (ps<0.05). We had decided *a priori* to assess the effect of PQ separately in each genotype because we expected a different response in the SVCT2-Tg mice, and this was performed with Bonferroni-corrected pairwise comparisons. PQ-treated SVCT2-Tg mice had lower SVCT2 expression than saline treated mice in liver, kidney and lung (ps<0.05; Fig. 7D, F & H), but the same effect was not seen in the wild-type mice (ps>0.43).

**Figure 8:**
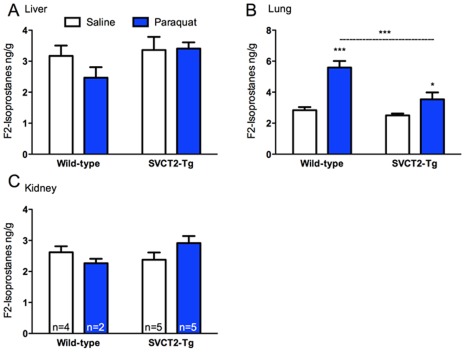
Oxidative stress following treatment with paraquat. F_2_-Isoprostanes were quantified as a marker for oxidative stress following paraquat treatment. F_2_-isoprostanes did not change according to paraquat treatment in (A) liver, or (C) kidney but were significantly elevated in (B) lung. Oxidative damage in lung was attenuated in SVCT2-Tg mice. *, *** p<0.05, 0.001 different from saline-treated control or as marked. Data shown are mean+S.E.M for wild-type and SVCT2-Tg mice treated with saline (white bars) and paraquat (blue bars). N = 5 mice per group, or as marked.

Oxidative stress in response to paraquat damage was assessed in the organs typically most affected by PQ treatment, lung and kidney, and also in the liver, which is responsible for metabolizing the drug. In the liver there was no increase in F_2_-isoprostanes following PQ treatment (p = 0.34), but there was also no difference according to genotype (p = 0.11; Fig. 8A). Expected increases in F_2_-isoprostanes were seen in lungs from PQ-treated mice (p<0.001). Bonferroni corrected pairwise comparisons were conducted following a significant Treatment×Genotype interaction from the omnibus ANOVA (p<0.05). Although PQ treatment increased F_2_-isoprostanes in both genotypes, the magnitude of the effect was much greater in wild-type (p<0.001) than SVCT2-Tg mice (p<0.05). Additional copies of SVCT2 did not affect F_2_-isoprostanes in saline-treated mice, but conferred significant protection in the case of PQ treatment (p<0.001; Fig. 8B). A small, but not significant, increase in F_2_-isoprostanes was observed in kidney from SVCT2-Tg mice (p = 0.097; Fig. 8C). Lower numbers of PQ-treated wild-type samples mean that it is difficult to interpret these data but overall there were no main effects of treatment or genotype in the kidney, and no interaction between the factors (ps>0.09).

## Discussion

The data we present demonstrates successful generation of a new mouse model that expresses functional additional copies of the SVCT2. Incorporation of the transgene in the founder line was shown by FISH to be in chromosome 9, rather than the native gene that is found in chromosome 2. The strong relation between the elevated mRNA expression in all tissues assayed, and ASC levels in all tissues except liver, demonstrate that the transgene led to functional copies of the transporter. This was further confirmed by the rescue of the lethal SVCT2(−/−) genotype in breeding studies. There was no detectable change in SVCT1 mRNA expression indicating a lack of compensatory mechanisms. However, we did not measure SVCT1 protein levels and nor did we assess transporter activity for SVCT1 and SVCT2 and thus it is possible that there are compensatory mechanisms that were not reported here. We did not measure gulonolactone oxidase activity, but it is unlikely that there was any large change in ASC synthesis because neither serum ASC levels, nor liver ASC levels differed between the groups.

Urinary ASC concentrations were similar in SVCT2-Tg and wild-type mice, suggesting that renal reabsorption of ASC was not affected. Early reports about the distribution of SVCT2 did not include the kidney [1] or indicated that it was present in very low levels [3]. The two-fold higher amounts of ASC observed in the kidney of SVCT2-Tg mice fit with strong mRNA and protein signals in both SVCT2-Tg and wild-type mice. Immunohistological staining of kidneys also revealed a strong signal for SVCT2 in both wild-type and SVCT2-Tg mice in a similar distribution. This immunostaining occurred in areas of the kidney outside the glomeruli, since it did not co-localize with von Willibrand factor. Absence of the SVCT2 in glomerular endothelial cells is perhaps surprising, since in most other vascular beds excepting the blood-brain barrier, ASC is highly expressed in endothelial cells [3], [19]. However, even in these beds, ASC is transferred from the blood to tissues by going around and not through the endothelial cells [20]. This, and the fenestrated nature of the renal glomerular endothelium, suggests that ASC movement from blood to urine is not retarded by the endothelial cells. Rather, the presence of the SVCT2 in what are likely tubular epithelial cells mirrors that of the SVCT1, which is needed for ASC reabsorption from the urine [6]. Although further studies of cellular localization of the SVCT2 in the kidney are needed, the non-glomerular location of SVCT2 could reflect a situation similar to that found in intestinal epithelia, in which the SVCT1 is localized to the brush border and SVCT2 to the basolateral border of the cells [21].

Although both wild-type and SVCT2-Tg mice were within the normal range for albumin in the urine, the lower levels of albumin in the urine from SVCT2-Tg mice suggests potentially improved kidney function in the SVCT2-Tg mice. There is evidence that ASC can decrease albuminuria in kidney diseases associated with oxidative stress (e.g. diabetes [22]) and our results suggest that this may be linked to function of the SVCT2.

In SVCT2(+/−) mice lower ASC levels are associated with neuromuscular weakness linked to hypomyelination and decreased collagen of sciatic nerves [23]. Muscular weaknesses and oxidative stress have also been seen in gulo(−/−) mice with decreased ASC levels [24]. We sought also to document any other functional changes resulting from the increase in ASC. Behaviorally the mice were normal with no change in exploratory or anxiety behaviors and no deficits or advantage in sensorimotor skills.

In initial studies there was an indication that SVCT2-Tg mice may be under elevated oxidative stress in liver as measured by F_2_-isoprostanes. This result could have offered a partial explanation for the lack of increase in ASC in this organ, but the result was not duplicated in the saline treated SVCT2-Tg mice in the PQ study. On the contrary, additional copies of the SVCT2-Tg were protective against oxidative stress in the lung induced by PQ treatments. Daily doses of PQ in rats caused specific and severe damage that was greatly reduced by concomitant administration of ASC when both were given intraperitoneally [17]. Oral vitamin C also limited acute kidney damage in patients exposed to acute PQ poisoning [25]. For the present study we chose a low dose of PQ and treated for just 6 days out of 21 to generate a mild oxidative stressor. This treatment schedule likely accounts for the lack of damage observed in the kidneys in these mice, particularly given the ability of all of the mice in the study to synthesize their own ASC in order to help offset such an oxidative stress, even in wild-type mice. In addition, the background strain, C57Bl6, is particularly resistant to kidney damage [26]. PQ exposure is particularly damaging to lung. This is partly because exposure is often by inhalation, but also because PQ accumulates in the lung and thus causes local damage through redox cycling and production of reactive oxygen species [27]. In wild-type mice PQ induced a large increase in F_2_-isoprostanes that was substantially decreased in the SVCT2-Tg mice. It is likely that the additional ASC available in the SVCT2-Tg mice helped to combat this increase in reactive oxygen species. Lung ASC levels were not significantly lowered by PQ treatment in the SVCT2-Tg mice but the SVCT2-Tg mice had a 1.5-fold increase in ASC over wild-type mice.

One of the most intriguing findings in this study was that mRNA signal was significantly decreased in the PQ treated mice in lung, kidney and liver, but not brain. In the case of lung, SVCT2-mRNA expression did not differ from wild-type although ASC levels were higher. The same result was not seen in the cortex arguing against the idea that the PQ mouse group happened by chance to have lower expression than the saline treated controls. Had this been the case then ASC levels would likely have been lower as well as suggested by correlative data from initial experiments in untreated mice. These intriguing data suggest a complicated response of the SVCT2 to oxidative stress that may be linked to changes in both function and amount of the transporter. SVCT2 has been shown to develop in culture in cortical endothelial cells [28], to be regulated by antioxidant status in C2C12 myotubules [29] and to change in response to low ASC in osteoblasts [30], and in liver but not cortex in *in vivo* studies in gulo(−/−) mice [10]. We repeatedly observe that brain SVCT2 levels are more tightly controlled than peripheral tissues, reinforcing the importance of ASC in the brain. This complex response pattern is clearly an area that deserves additional study.

We propose that this mouse will be of use in studies that require increases of ASC at the cellular level. Supplementation of mice with oral ASC, particularly wild-type mice that can synthesize their own ASC, does not lead to long-term increases in tissue ASC, and particularly not in the brain, which is under tight regulation. This new model can therefore be of use in disease models, and to study the role of ASC in response to pharmacological agents. The mouse may be particularly useful in studies of lung and kidney disease in which tissues showed particularly large increases in ASC. By crossing different models together it will also be possible to investigate further the role of high, and in combination with the SVCT2(+/−) mouse, low ASC in diseases with a genetic basis.
