# Correction: Increased Expression of SVCT2 in a New Mouse Model Raises Ascorbic Acid in Tissues and Protects against Paraquat-Induced Oxidative Damage in Lung

**DOI:** 10.1371/annotation/31991586-54ab-41ff-8092-fd92989e97e6

**Published:** 2012-05-15

**Authors:** Fiona Edith Harrison, Jennifer Lee Best, Martha Elizabeth Meredith, Clare Ruth Gamlin, Dorin-Bogdan Borza, James Michael May

There was an error in the sixth author's name. The correct name is James Marion May. 

